# Breaking the Silence: A Community-Based Cross-Sectional Study Exploring the Attitude Towards Premenstrual Syndrome Among Adults in Perambalur District of Tamil Nadu, India

**DOI:** 10.7759/cureus.76847

**Published:** 2025-01-03

**Authors:** Vijay Anand V, Neeraj V Mohandas, C. Brilly Swarna, K. Caroline Singh, Abishek Jeevagan, Sakthiyasree S. V., Samhitha P. G., Samyuktha S. S., S. Santhosh Kumar, Sasidharan S. G.

**Affiliations:** 1 Community Medicine, Dhanalakshmi Srinivasan Medical College and Hospital, Siruvachur, Perambalur, IND

**Keywords:** attitude, knowledge, menstrual health, menstrual pain, premenstrual syndrome(pms)

## Abstract

Background

Menstruation is a natural process crucial for female reproductive health. Premenstrual syndrome (PMS) is characterized by physical and emotional symptoms occurring before menstruation. Social and cultural factors significantly influence menstrual attitudes and PMS perception. Cultural norms and a lack of confidence during this phase may impede women's ability to lead innovation. This is where men can play a crucial role in driving change by actively engaging in menstrual health advocacy. By challenging restrictive practices and negative perceptions surrounding menstruation, men can contribute to better menstrual hygiene management. The objective of this study was to determine the independent predictors of attitude towards PMS among both male and female adult populations in Perambalur district, Tamil Nadu, India.

Materials and methods

A community-based cross-sectional study was conducted from August 2024 to October 2024. A pre-tested, structured questionnaire was utilized to assess the attitude towards PMS. The questions on attitude towards PMS were scored as per the recommendation of an expert committee. The data were entered into Microsoft Excel, numerically coded, and analyzed using IBM SPSS version 26. Descriptive statistics was used to characterize the study participants and was expressed in frequencies, percentages, and mean (±standard deviation (SD)). Independent samples t-test and one-way ANOVA were used to determine the significant difference between the mean attitude score and the independent variables. A multivariable linear regression model was used to determine the independent predictors of attitude score. A p-value <0.05 was considered statistically significant.

Results

The study included a total of 281 study participants. The mean (±SD) age of the study participants' age was 32.21 (±8.64) years. The mean (±SD) attitude score among the study participants was 7.12 (±2.17). The independent predictors, which had a positive impact on attitude score, were as follows: (1) age groups (18-25 years, 26-35 years, and 36-45 years), (2) male gender (B = 0.77; 95% CI 0.28-1.27), (3) school education (B = 0.74; 95% CI 0.21-1.28). The independent predictor, which had a negative impact on attitude scores, was a rural area of residence (B = -0.53; 95% CI -1.07 to -0.26).

Conclusion

The attitude towards PMS is multifaceted. Interventions should address the gaps through multi-pronged approaches, including education, community awareness, and policy changes, while also considering broader social and environmental factors impacting menstrual health.

## Introduction

Menstruation is a fundamental physiological process in every woman’s life, and it is necessary for the uterine lining renewal to prepare it for potential pregnancy [1]. Menstrual health is an integral aspect of overall women's health, significantly impacting their physical, mental, and social well-being [2]. Normal menstruation is defined as cyclic bleeding from the uterine corpus between menarche and menopause [3].

Premenstrual syndrome (PMS) refers to a cluster of physical, behavioural, and emotional symptoms occurring during the luteal phase, typically the week before menstruation [4]. These symptoms, often commencing after the 13th day of the menstrual cycle, can often disrupt women's daily lives [1]. The prevalence of PMS varies across countries with estimates in India ranging from 14.3% to 74,4% [5,6]. The Diagnostic and Statistical Manual of Mental Disorders-5 (DSM-5) classifies PMS by physical symptoms (abdominal bloating, pain, breast tenderness, joint/muscle ache, headache, swelling, and weight gain) and behavioural symptoms (anger, irritability, low mood, anxiety, confusion, and social withdrawal) [7]. While various theories exist regarding PMS aetiology, most remain scientifically unproven [8,9].

The attitudes and beliefs concerning menstruation are influenced by a person’s immediate family environment and by the broader cultural influences [10]. Menstrual attitudes are considered a multifaceted social construct [11]. Menstrual socialization and early exposure to attitudes towards menstruation significantly influence individual perspectives [12]. Despite the physiological similarities of menstruation worldwide, perceived experiences can vary considerably due to differing sociocultural frameworks of understanding [11]. Studies conducted in similar settings have also demonstrated a link between religious beliefs, culture, and PMS [13,14].

India, a diverse nation with multiple religious and ethnic groups, possesses unique cultures, customs, and practices [15]. However, menstruation and menstrual practices are still often shrouded in taboos and sociocultural restrictions throughout the country [16]. In some rural areas of Tamil Nadu, the practice of isolating women from participating in day-to-day activities during the period of menstruation still exists because menstruation is considered religiously impure and ceremonially unclean [17]. This leads to misconceptions and harmful practices, hindering open discussions and potentially resulting in negative health outcomes [16]. Consequently, culture significantly influences the perception and experience of PMS symptoms [18].

In today's world, where women increasingly pursue higher education and professional careers, understanding male perspectives on their partner's physiological and hormonal changes during PMS is crucial [19]. Cultural norms and societal expectations can significantly impact women's confidence during the premenstrual phase, potentially hindering their ability to fully participate in and lead innovation within their communities [20]. This presents a significant opportunity for men to drive a positive change within the realm of menstrual health. By actively engaging in advocacy efforts, men can play a crucial role in challenging restrictive cultural practices and dismantling deeply ingrained negative perceptions surrounding menstruation [21]. Furthermore, by actively participating in the discourse surrounding menstrual health, men can contribute significantly to improving menstrual hygiene management practices, thereby enhancing the overall well-being of women and fostering greater gender equity within the community [21]. Mutual support during this period can help alleviate distress and improve communication within couples, which is essential for a fulfilling marital life [22,23].

Comprehensive education, positive attitudes, and accurate reproductive health knowledge among both men and women are vital in combating gender bias and stigma surrounding menstrual health [24]. This community-based cross-sectional study was conducted among both adult males and females in the Perambalur district of Tamil Nadu, India, to determine the independent predictors of attitude towards PMS.

## Materials and methods

Study setting

A community-based cross-sectional study was conducted among both male and female adults in the field practice area of a medical school in the Perambalur district of Tamil Nadu, India, from August 2024 to October 2024. Ethical clearance (IECHS/IRCHS/No:503, March 26, 2024) was obtained from the Institutional Ethical Committee of Dhanalakshmi Srinivasan Medical College and Hospital prior to the commencement of the study.

Inclusion and exclusion criteria

All adult males and females between the ages of 18-60 years were included in the study. Critically ill or comatose patients who were unable to answer the questions in the questionnaire were excluded.

Sample size determination

The sample size was calculated from the proportion of participants who had adequate attitudes regarding pre-menstrual symptoms (61.1%) from the study by Wong et al. [25]. The formula used was: 

n = (Z_(1-α/2)^2 * P * Q)/d^2

[ Z1-α/2 = 1.96, P (prevalence) = 61.1, Q (100-prevalence) = 38.9, d (relative precision) = 10%] and the minimum sample size with 80% power, 95% confidence interval, and 15% non-response rate came up to 281.

Sampling technique and data collection

The field practice area of the only medical school in Perambalur district, Tamil Nadu, India, covered 18 villages. Two villages were selected by simple random sampling considering limitations in resources and manpower. Population proportional to size sampling was used to decide the number of participants to be selected from each village. A simple random sampling was done using the line list of the households in each village to select the participants to be included in the study. The study personnel conducted house-to-house visits, and participants were enrolled as per the inclusion criteria after obtaining informed consent. If the participants were not available, the eligible participants of the next household, as per the line list, were enrolled in the study.

Data collection was conducted using a pre-tested, structured questionnaire. A draft questionnaire was developed in consultation with a distinguished panel of experts using a mini-modified Delphi consensus method [26]. Subsequently, a pilot study involving 20 participants was conducted to assess the clarity, comprehensiveness, and feasibility of the instrument. These 20 participants were subsequently excluded from the primary data analysis. Based on the feedback from the pilot study, necessary modifications were incorporated into the final version of the questionnaire. To ensure the internal consistency of the study tool, Cronbach's alpha coefficient was calculated, yielding a satisfactory score of 0.72, indicating an acceptable degree of internal consistency among the items within the questionnaire.

The questionnaire comprised two separate sections. The first section focused on the sociodemographic characteristics of the study participants which included area of residence, education, occupation, and per capita family income. To ascertain the socioeconomic status of the study participants, a modified B.G. Prasad scale was employed [27]. The second section encompassed a series of 13 questions designed to comprehensively assess the attitudes of the study participants towards PMS.

A team of experienced public health specialists, psychiatrists, and gynaecologists were consulted, and as per consensus, each item in the questionnaire was scored. The answers having a positive attitude were given a score of “1” and those having a negative attitude were given a score of “0”. The total scores ranged from 0 to 13. The final questionnaire is included in the appendices.

Statistical analysis

The data collected were entered into Microsoft Excel (Microsoft Corp., USA), numerically coded, and analyzed using IBM SPSS Statistics for Windows, version 26.0 (released 2019, IBM Corp., Armonk, NY). Descriptive analysis was conducted to characterize the study population and was expressed in frequencies, percentages as well as mean (±standard deviation (SD)). The Kolmogorov-Smirnov test was used to assess the normality of data distribution. Bivariate analysis was conducted using independent samples t-test and one-way ANOVA. Following one-way ANOVA, post-hoc Tukey tests were conducted to pinpoint independent variables exhibiting statistically significant disparities with the mean attitude score. Pearson’s correlation coefficient was used to assess the correlation of age and per capita income with the attitude score.

To identify the statistically significant independent predictors of attitude scores, a multivariable linear regression model was utilized. All variables which demonstrated a statistical significance (p < 0.05) in bivariate analyses were taken for the multivariable linear regression. The adjusted R-squared value was calculated to quantify the proportion of variance in the total awareness score that could be attributed to the identified independent predictors. Furthermore, the Durbin-Watson statistic was utilized to assess the presence of autocorrelation within the data. Unstandardized B coefficients were calculated to quantify the magnitude of change in the attitude score associated with a unit change in each independent variable. A p-value <0.05 was considered statistically significant.

## Results

The mean (±SD) age of the 281 study participants was 32.21 (±8.64) years. The majority of the study participants (108 (38.4%)) were aged between 26 and 35 years, and only 27 (9.6%) were aged above 45 years. Most of the participants were females (168 (59.8%)). The basic characteristics of the study participants are given in Table 1.

**Table 1:** Sociodemographic characteristics of the study participants (N = 281)

Basic characteristics		Frequency (n)	Percentage (%)
Age (in years)	18-25	80	28.5
	26-35	108	38.4
	36-45	66	23.5
	>45	27	9.6
Gender	Male	113	40.2
	Female	168	59.8
Education	School education	144	51.2
	Graduation and above	137	48.8
Occupation	Employed	255	90.7
	Unemployed	26	9.3
Marital status	Married	192	68.3
	Unmarried	78	27.8
	Divorced/Separated/ Widowed	11	3.9
Area of Residence	Urban	124	44.1
	Rural	157	55.9
Socio-economic status (as per the modified B. G. Prasad scale)	Upper and Upper Middle class	202	71.9
	Middle and Lower Middle class	62	22.1
	Lower class	17	6.0

**Figure 1:**
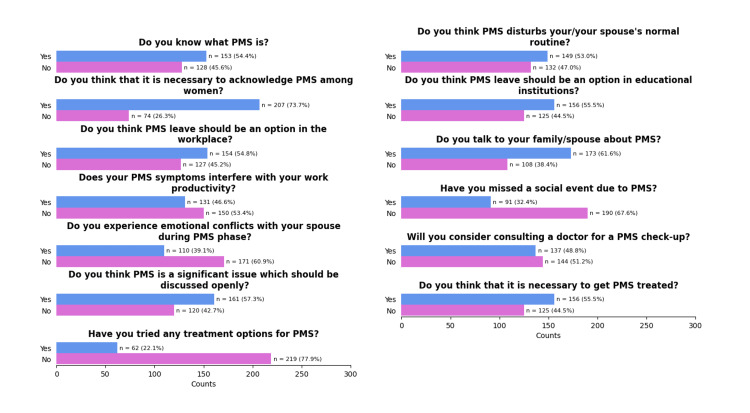
Responses of the study participants to the questions on attitude towards premenstrual syndrome (PMS) (N = 281)

Figure 1 provides a graphical representation of the study participants' responses to the 13 questions designed to assess their attitudes toward PMS. The majority (153 (54.4%)) of the study participants knew what PMS was, and most of them (161 (57.3%)) felt that PMS is a significant issue that should be discussed openly. The majority (156 (55.5%)) felt that it is necessary to get PMS treated.

**Figure 2:**
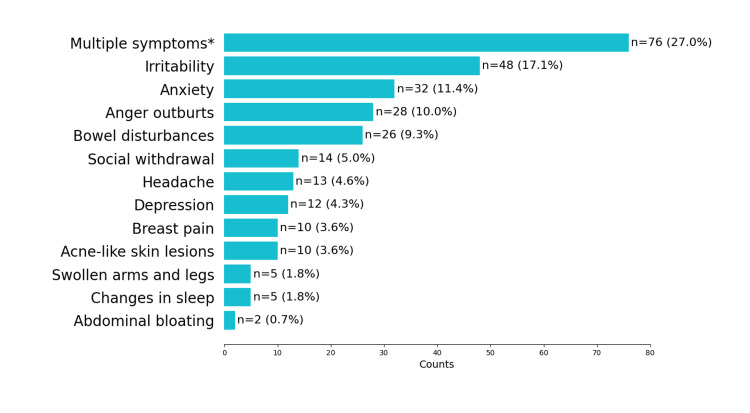
Symptoms recalled by the study participants as part of premenstrual syndrome (PMS) (N = 281) *combination of different individual symptoms

Figure 2 represents the most common symptoms recalled by the study participants as part of PMS. The majority (76 (27%)) felt that multiple symptoms, including irritability, anxiety, anger outbursts, bowel disturbances, and social withdrawal, are experienced during PMS.

The mean (±SD) attitude score of the study participants regarding PMS was 7.12 (±2.17). A statistically significant difference was found between the mean attitude score and gender, area of residence, and education of the study participants. The details are given in Table 2.

**Table 2:** Difference in the mean attitude score of the study participants based on the sociodemographic characteristics (N = 281) *p-value <0.05 is statistically significant. Independent samples t-test was used.

Basic characteristics		Mean attitude score (±SD)	p-value
Gender	Male	6.63 (±2.15)	0.002*
	Female	7.45 (±2.12)	
Occupation	Employed	7.06 (±2.20)	0.16
	Unemployed	7.69 (±1.78)	
Area of residence	Urban	7.58 (±2.07)	0.001*
	Rural	6.75 (±2.18)	
Education	School education	6.63 (±2.15)	<0.001*
	Graduation and above	7.64 (±2.07)	

Table 3 presents the results of the one-way ANOVA test used. A statistically significant difference was found between the mean attitude score and the age of the study participants. A post-hoc Tukey test showed a statistically significant difference in the mean attitude score among the study participants having age (in years) >45-18 to 25 years (mean difference 1.59), 26 to 35 years (mean difference 1.26), and 36 to 45 years (mean difference 1.40).

**Table 3:** Difference in the mean attitude score of the study participants based on specific characteristics (N = 281) *p-value <0.05 is statistically significant. A one-way ANOVA test was used. †Statistically significant difference in the post-hoc Tukey test. Age (in years): >45-18 to 25 (mean difference 1.59), 26 to 35 (mean difference 1.26), 36 to 45 (mean difference 1.40)

Basic characteristics		Mean attitude score (±SD)	p-value
Age (in years)	18-25	7.45 (±2.09)	0.01*
	26-35	7.12 (±2.11)	
	36-45	7.25 (±2.17)	
	>45†	5.85 (±2.28)	
Socio-economic status	Upper and Upper Middle class	7.12 (±2.24)	0.61
	Middle and Lower Middle class	7.00 (±2.08)	
	Lower class	7.58 (±1.46)	
Marital status	Married	6.97 (±2.13)	0.10
	Unmarried	7.55 (±2.16)	
	Divorced/Separated/ Widowed	6.63 (±2.65)	

Correlates of the attitude score

The Pearson correlation revealed a statistically significant positive correlation between the age and attitude score (r = 0.14, p-value = 0.016). No statistically significant correlation was found between the per capita income and attitude score (r = 0.05, p-value = 0.40).

Regression analysis

The attitude score was regressed on predicting variables that were statistically significant in the bivariate analysis (age (in years), gender, area of residence, and education). The overall regression model was statistically significant, and the independent variables significantly predict attitude score, F(3, 277) = 9.80, p-value < 0.001. It showed an R-square value of 0.56 and an adjusted R-square value of 0.43, which implies that age (in years), gender, area of residence, and education accounted for 43% of the variation in the attitude score. The Durbin-Watson statistic was 2.05, indicating no autocorrection.

In this study, the statistically significant positive predictors of the attitude score were as follows: (1) age: (a) 18-25 years (B = 1.59, t-value = 3.35, p-value = 0.001), (b) 26-35 years (B = 1.26, t-value = 2.75, p-value = 0.006), (c) 36-45 years (B = 1.40, t-value = 2.87, p-value = 0.004); (2) male gender (B = 0.77, t-value = 3.07, p-value = 0.002); (3) school education (B = 0.74, t-value = 2.74, p-value = 0.006). The statistically significant negative predictor of the attitude score was the area of residence: rural (B = -0.53, t-value = 1.95, p-value = 0.04). The results are presented in Table 4.

**Table 4:** Independent predictors of the attitude score *p-value <0.05 is statistically significant. Multivariable linear regression was used.

Variables		Unstandardized beta coefficient (95% CI)	Standardized beta coefficient	t-value	p-value
Age (in years)	18-25	1.59 (0.66-2.53)*	0.33	3.35	0.001*
	26-35	1.26 (0.36-2.17)*	0.28	2.75	0.006*
	36-45	1.40 (0.44-2.36)*	0.27	2.87	0.004*
	>45	1			
Gender	Male	0.77 (0.28-1.27)*	0.17	3.07	0.002*
	Female	1			
Area of residence	Rural	-0.53 (-1.07 to -0.26)*	-1.12	-1.95	0.041*
	Urban	1			
Education	School education	0.74 (0.21-1.28)*	1.17	2.74	0.006*
	Graduation and above	1			

## Discussion

This study provides an overview of the attitude towards PMS among adult men and women in the Perambalur district of Tamil Nadu, India. It identifies the independent predictors associated with attitude, offering valuable insights for improving the outlook towards the reproductive health of women.

In this study, the mean (±SD) attitude score among the study participants was 7.12 (±2.17). The study conducted in Norway by Kvalem et al. reported a mean (±SD) score of 4.40 (±1.26), which indicated that the average woman considered menstruation to be more bothersome [28]. Siddique et al. have reported a high mean attitude score of 12.58( ±1.57) (out of 14) among women of reproductive age in Bangladesh [29]. However, Hoerster et al. have reported that Indian women scored significantly higher than American women using the Menstrual Attitude Questionnaire [30]. These differences in menstrual attitudes seen in different studies are due to the different study tools used.

Age was found to be a statistically significant positive predictor of attitude towards PMS in this study. Similar results were reported by Siddique et al. from Bangladesh [29]. Pedro et al. also reported a statistically significant correlation between age and severity of PMS [31]. Logue et al. observed that younger women experience more menstrual symptoms, while older women experience more premenstrual symptoms [32]. Such differentiation may explain the variability across studies. However, this study did not explore this aspect specifically. Pedro et al. observed that women in the younger age group (<40 years) were more willing to explore treatment options as compared to those above 40 years. In this study, younger women (<40 years) had higher odds to explore treatment options for PMS but it was not found to be statistically significant.

Previous research focusing on women reported that men were insensitive with regard to PMS [33]. Aggarwal et al. from India reported that knowledge of PMS is comparable among Indian females and males and that a major factor regarding increased awareness among males is the willingness of females to discuss their PMS symptoms with their male partners [34]. In this study, male gender was found to be a statistically significant positive predictor of awareness regarding PMS and 61.6% of the participants were likely to talk to their partners regarding PMS. This shows the changing mindset of the current generation with regard to PMS.

In this study, residence in rural areas was found to be a statistically significant negative predictor of awareness, which is consistent with the results of the study by Aggarwal et al. [34]. The urban-rural differences in the attitudes towards premenstrual and menstrual symptoms are due to the differences in culture surrounding menstruation in urban and rural areas [35]. Wong et al. reported that although the awareness regarding menstrual symptoms was less among rural women, urban women reported more severe symptoms probably due to the higher pain endurance of rural women [36].

The positive effect of education on health awareness and personal health is a well-known fact [37]. School education was found to be a statistically significant positive predictor of PMS awareness in this study. Ahmed et al. from Egypt reported that after the implementation of an educational intervention, awareness regarding PMS among adolescents improved significantly [38]. Abdalla et al. also reported similar results after implementing an educational programme [39]. Implementing menstrual hygiene awareness to both boys and girls right from the school level is very crucial; however, Sharma et al. reported that menstrual health promotion in schools remains an issue of concern in India [40].

A key strength of this study lies in its inclusive design, encompassing both male and female perspectives. Furthermore, the research employed a robust methodological framework, including a pre-tested questionnaire and regression models for in-depth data analysis. However, several limitations warrant consideration. The focus on the field practice area of a single medical institution in the district may limit the generalizability of the findings to the broader population. The reliance on self-reported data through questionnaires raises concerns regarding potential recall bias and social desirability bias, potentially impacting the accuracy of responses. A validated questionnaire was not used in this study; however, the internal consistency was assessed using Cronbach’s alpha which was found to be acceptable. Finally, the cross-sectional design of this study precludes the establishment of definitive causal relationships between variables.

Way forward

Cultivating a positive societal perspective towards menstrual health is paramount. Early-stage education, encompassing both male and female students, has the potential to foster a more accepting and informed attitude towards PMS. At the community level, parental education, with a particular emphasis on mothers, is crucial. This education should aim to dispel prevalent myths and equip parents with accurate information, thereby empowering them to effectively educate their children. Given the current underrepresentation of menstrual health within school curricula, formal classroom instruction on this vital topic is urgently required.

## Conclusions

This study concluded that the attitude of this population towards PMS is multifaceted and ambivalent in nature. Demographic factors, including age, gender, place of residence, and educational attainment, were observed to exert a statistically significant influence on these attitudes. Interventions aimed at improving attitudes towards PMS should encompass a multi-pronged approach. This includes the implementation of school-based educational programs, community-based awareness campaigns, and supportive policy frameworks. Furthermore, it is crucial to recognize that these interventions should not only be limited to individual health education. Addressing the broader social and environmental factors that significantly impact menstrual health is equally essential.

## Appendices

**Table 5:** Questionnaire

Breaking the Silence: A Community-Based Cross-Sectional Study Exploring the Attitude Towards Premenstrual Syndrome Among Adults in Perambalur District of Tamil Nadu, India	
Section 1: Sociodemographic Profile	
1. Age:	
2. Sex:	a. Male b. Female
3. Education:	a. Illiterate b. Primary (1-4) c. Middle (5-7) d. High School (8-10) e. Higher Secondary (11-12) f. Graduation g. Postgraduation
4. Occupation:	a.Professional b.Homemaker c.Skilled d.Unskilled e.Unemployed f. Retired g. Others(specify):
5. Marital status:	a.Unmarried b.Married c.Legally Divorced d. Living in separation e. Widow
6. Area of Residence:	a. Rural b. Urban
7. Family income per month (in INR):	
8. Total number of members in the family:	
Section 2: Questions on Attitude regarding Pre-Menstrual Syndrome (PMS)	
1. Do you know what Pre-Menstrual Syndrome (PMS) is?	a. Yes b. No
2. Do you think Pre-Menstrual Syndrome (PMS) disturbs you/your spouse’s normal routine?	a. Yes b. No
3. Do you think it is necessary to acknowledge Pre-Menstrual Syndrome (PMS) among women?	a. Yes b. No
4. Do you think Pre-Menstrual Syndrome (PMS) leave should be an option in educational institutions?	a. Yes b. No
5. Do you think Pre-Menstrual Syndrome (PMS) leave should be an option in the workplace?	a. Yes b. No
6. Do you talk to your family/spouse about Pre-Menstrual Syndrome (PMS)?	a. Yes b. No
7. Does your/your spouse’s Pre-Menstrual Syndrome (PMS) symptoms interfere with your work productivity?	a. Yes b. No
8. Have you/your spouse missed a social event due to Pre-Menstrual Syndrome (PMS)?	a. Yes b. No
9. Do you experience emotional conflicts with your spouse during Pre-Menstrual Syndrome (PMS) phase?	a. Yes b. No
10. Do you think Pre-Menstrual Syndrome (PMS) is a significant issue to be discussed openly?	a. Yes b. No
11. Will you/your spouse consider consulting a doctor for Pre-Menstrual Syndrome (PMS) check up?	a. Yes b. No
12. Do you think it is necessary to get Pre-Menstrual Syndrome (PMS) treated?	a. Yes b. No
13. Have you/your spouse tried any treatment options for Pre-Menstrual Syndrome (PMS)?	a. Yes b. No
